# University–industry R&D linkage metrics: validity and applicability in world university rankings

**DOI:** 10.1007/s11192-016-2098-8

**Published:** 2016-08-13

**Authors:** Robert J. W. Tijssen, Alfredo Yegros-Yegros, Jos J. Winnink

**Affiliations:** 1 Centre for Science and Technology Studies (CWTS), Leiden University, PO Box 905, 2300 AX Leiden, The Netherlands; 2 DST-NRF Center of Excellence in Scientometrics and Science, Technology and Innovation Policy, Stellenbosch University, Stellenbosch, South Africa; 3 NL Patent Office, The Hague, The Netherlands

**Keywords:** University rankings, Research performance, Indicators, Measurement

## Abstract

In September 2015 Thomson Reuters published its *Ranking of Innovative Universities* (RIU). Covering 100 large research-intensive universities worldwide, Stanford University came in first, MIT was second and Harvard in third position. But how meaningful is this outcome? In this paper we will take a critical view from a methodological perspective. We focus our attention on the various types of metrics available, whether or not data redundancies are addressed, and if metrics should be assembled into a single composite overall score or not. We address these issues in some detail by emphasizing one metric in particular: university–industry co-authored publications (UICs). We compare the RIU with three variants of our own *University*–*Industry R&D Linkage Index*, which we derived from the bibliometric analysis of 750 research universities worldwide. Our findings highlight conceptual and methodological problems with UIC-based data, as well as computational weaknesses such university ranking systems. Avoiding choices between size-dependent or independent metrics, and between single-metrics and multi-metrics systems, we recommend an alternative ‘scoreboard’ approach: (1) without weighing systems of metrics and composite scores; (2) computational procedures and information sources are made more transparent; (3) size-dependent metrics are kept separate from size-independent metrics; (4) UIC metrics are selected according to the type of proximity relationship between universities and industry.

## Introduction

This paper takes a closer look at various measurements to describe and analyze R&D linkages between universities and industry. Our international perspective is that of world university rankings, which have become increasingly popular in recent years as a platform for institutional performance analysis and international benchmarking. There are some 17,000 higher education institutions in the world (Rauhvargers 2011), including thousands of research-intensive universities each addressing a variety of local societal needs and often engaged in global activities. On top of research and teaching, they are also expected to engage more with society and reach out to the business sector and industry. Today many research universities worldwide are also held accountable for their ‘third mission’ activities and achievements. Given current government policies that strongly promote synergies between high-quality science and business sector innovation, there is a great need for effective analytics and valid metrics to monitor and assess connections between public science and private R&D.

Any systematic attempt to identify, classify or measure the performance of universities in terms of their institutional linkages with the business sector is analytically relevant. Evermore universities are engaged in commercialization of research and transfer of research outputs and technologies to the business sector. Interestingly, links between academic science and technological innovation in general, and university–industry research connections in particular, are not well understood—let alone adequately represented in world university ranking systems. These rankings fill an information gap within an academic culture of increasingly fierce global competition and status seeking among research-intensive universities. Rising in prominence as an information tool they have affected institutional missions and functions of many universities and colleges worldwide (Marginson 2007).

However, rankings are reductionist information tools as they present only parts of complex phenomena; they disproportionately value those features that are measurable with currently available international or institutional sources. Moreover, several high-profile ranking systems apply a weighted composite of individual indicators and metrics to create a single ‘index score’ of university performance and present league tables that emphasize these final scores. The selection criteria for choosing the constituent metrics, how those measurements are normalized or weighted, and how they are finally added into an overall score are usually not explicit or well-documented. Nor are there any authoritative guidelines or ‘best practices’ on how metrics should be weighted and integrated into a ‘league table’ ranking system. This lack of transparency is generally seen as one of the major methodological shortcomings of such league tables and ‘black box’ ranking systems. Another weakness of many world ranking systems is their neglect of an institution’s size as an ordering principle—either in terms of inputs, activities or outputs—which not only hampers ‘like by like’ comparisons of similar-size universities but also rules out the possibility to incorporate and assess possible size-effects (such as ‘critical mass’ or ‘economies of scale’ benefits).

Recently, the information provider *Thomson Reuters* (TR) has entered in the rankings arena with of a *Ranking of Innovative Universities*—*RIU* (Thomson Reuters 2016a). In order to create this ranking, TR first pre-selected the 500 universities worldwide with the largest number of research articles in scholarly journals over the 2008–2015 period, and kept the institutions which filed 70 or more US patents during the same time-span. Those institutions that remained were ranked by 8 metrics.^1^ A composite score ranks the universities by summing the ranks for each metric, where the contributing metrics are weighted equally.^2^ From here on we will refer to these as *RIU* scores.^3^

Stanford University emerges as the world’s number 1 in the RIU; Massachusetts Institute of Technology is number two university, while Harvard University is third. These are indeed generally seen as leading ‘entrepreneurial’ universities whose institutional mission include extensive engagement with the business sector and creating economic activity (e.g. Guerrero and Urbano 2012). TR’s website provides no ranking information for separate metrics. Given the arbitrariness of their weighting system and the lack of an explicit rationale for implementing this particular selection of metrics, several methodological issues are left unanswered. One of which is the possible detrimental effect of overlaps or redundancies between various measures. Such data deficiencies are clearly a potential weakness in any multiple-metrics ranking system, one that needs to be addressed to ensure fair comparisons across universities.

How should one assess the practical relevance and information quality of such a ranking systems? Although some academic studies examined the statistical validity of indicators and relationships between metrics (Goldstein and Spiegelhalter 1996; Stella and Woodhouse 2006; Tofallis 2012), there is still no theory-based rationale for the choice of performance indicators nor authoritative guidelines on how within ranking systems show deal with highly related metrics.

In this paper we will take a critical and pragmatic view of this type of ranking from a broader methodological perspective. We focus our attention on the various types of metrics available, whether or not data redundancies are addressed, and if metrics should be assembled into a composite measure or kept separate. We address these issues in some detail by emphasizing one metric in particular: the share of *university*–*industry co*-*authored publications* (UICs for short) within a university’s total publication output. This is one the eight metrics in *RIU* but it also features in two other ranking systems: *Leiden Ranking* (www.leidenranking.com)^4^ and *U*-*Multirank* (www.umultirank.org).^5^

Our main research question is: are UIC-based metrics suitable for university rankings? Unpacking this question our cross-validation study addresses the following sequence of sub-questions:

Q1: What do UIC-based metrics represent, and how do they relate to other metrics of university ‘innovation’ and?
Q2: Should one apply size-dependent or size-independent UIC-based metrics?
Q3: What are the (dis)advantages when several metrics are combined or integrated into a single composite score?
Q4: Can one develop a ‘good practice’ to develop more informative university rankings?

## Studies of university–industry R&D linkages

A multitude of case studies provides insights into this complex of relationships between universities and the business sector; many focus their attention of variety and workings of knowledge transfer channels that may exist or occur, thereby highlighting some of the possible driving forces and determinants of R&D interactions (e.g. Arza 2010; Dutrénit et al. 2010; De Fuentes and Dutrient 2012; Ramos-Vielba and Fernández-Esquinas 2012; Perkmann et al. 2013). One of the main structural drivers and factors that shape university–industry R&D connections is ‘proximity’, which comes in many shapes and forms (Laursen et al. 2011; De Fuentes and Dutrénit 2014). Various empirical studies have shown that successful transfer of knowledge from universities to industry is shaped by geography; small distances tend to have positive effects on a firm’s innovation performance (e.g. Audretsch and Feldman 1996). Geographical proximity is an important factor in university–industry R&D linkages (e.g. Ponds et al. 2007; Bjerregaard 2010), where distance from the university decreases the likelihood that a firm collaborates with the university (e.g. Laursen et al. 2011; Hong and Su 2013).

Proximity offers a rich conceptual framework and a sophisticated analytical framework for assessments of university performance profiles. Imposing a classification system to its variety of manifestations, Boschma (2005) defined five main categories of proximity: geographical, cognitive, organizational, social, and institutional. Where ‘geographical proximity’ refers to the spatial or physical distance between partners, the notion of ‘cognitive proximity’, captures the degree to which people sharing the same knowledge base and expertise (often with complementarity sets of skills and competencies). While ‘social proximity’ is defined in terms of socially embedded relationships (friendship, shared past experience, behavioral codes, common culture, mutual trust), ‘institutional proximity’ is associated with similarities in terms of institutional frameworks and shared organizational arrangements. The latter comprises differences in terms of ‘organizational proximity’ regarding the degree of autonomy of partners.

Summarizing, proximities may cover both ‘stocks’ and ‘flows’. The former relates to financial capital, knowledge creation capacity, human capital and R&D infrastructures, while the latter refers to dynamic features such as institutional mobility, research collaboration, knowledge dissemination and utilization. Measurements of proximity related to either (dis)similarities in ‘stock profiles’ of connected R&D partners, or ‘flow profiles’ of the nature and magnitude of those connections. Both types of metrics may comprise a size-dependent and size independent variant. TR’s ranking system is not only a mix of stock-based metrics and flow-based metrics, but comprises both size-dependent and size-independent measures.

None of the above-mentioned case studies systematically analyze university–industry relationships according across the different types of proximities. However, three streams of literature can be identified, each analyzing a specific aspect of collaborative knowledge production in relation to proximity characteristics:

- The role of proximity in the choice of collaboration partners and research network formation (e.g. Autant-Bernard et al. 2007; Balland 2012);
- relationships between proximity to R&D partners and the innovative performance of the collaborating organisations (Nooteboom et al. 2007; Broekel and Boschma 2012);
- how proximity explains processes of knowledge production and knowledge sharing (Weterings and Ponds 2009).

In this paper we focus our attention on the last of these streams, thereby restricting our scope to ‘flows’-based proximity metrics. Ours is a descriptive study; we do not imply any causality in terms of unidirectional or bidirectional knowledge flows (i.e. from universities to industry or from science to technology, or vice versa).

## University–industry co-publications and performance measurement

The number of university–industry co-authored publications (UICs) produced by a university is one of several tangible outputs of productive university–business interactions, but the meaning of this statistic and its analytical significance, let alone the cause/effect relationship with other performance metrics such as industry income, is neither clear nor straightforward. Although the theoretical foundation for metrics-based ‘quantitative indicators’ to evaluate university–industry relationships was laid down in the 1990s (Bonaccorsi and Piccaluga 1994), most empirical case studies of UIC activity are of more recent date: Anderson and Dalpé (1992), Hicks et al. (1993), Hicks (2000), Calvert and Patel (2003), Butcher and Jeffrey (2005), Lundberg et al. (2006), Tijssen (2006), Glänzel and Schlemmer (2007), Ponds et al. (2007), Sun et al. (2007), Perkmann and Kathryn (2009), Klitkou et al. (2009), Tijssen et al. (2009), Levy et al. (2009), Abramo et al. (2009, 2010, 2011), Tijssen (2012), Wong and Singh (2013), Muscio et al. (2014), Fan et al. (2015).

In spite of the fact that UIC-based metrics are now used publicly to measure the relative strengths of university–industry research collaboration, the validity and added value of such measurements, and their scope for broader analytical applications, are still not well understood (Perkman et al. 2011; Tijssen 2012; Aldats and Fiegenbaum 2015). Clearly there are many other ways to study and measure university–industry linkages, cooperation and impacts (Healy et al. 2014), but UICs are currently the only available information source for large-scale and systematic quantitative analysis. The reliance on a single UIC-based metric within a university ranking system will inevitably introduce a limited view, thus posing a risk of biases and misuse. In view of the increasing relevance of university rankings for promotional and analytical applications, these usage issues have come to the fore in several critical reviews that were published in recent years (e.g. Rauhvargers 2011; Marope et al. 2013). Collectively these two sources capture most of the key issues and are an excellent entry point for further reading.

**Fig. 1:**
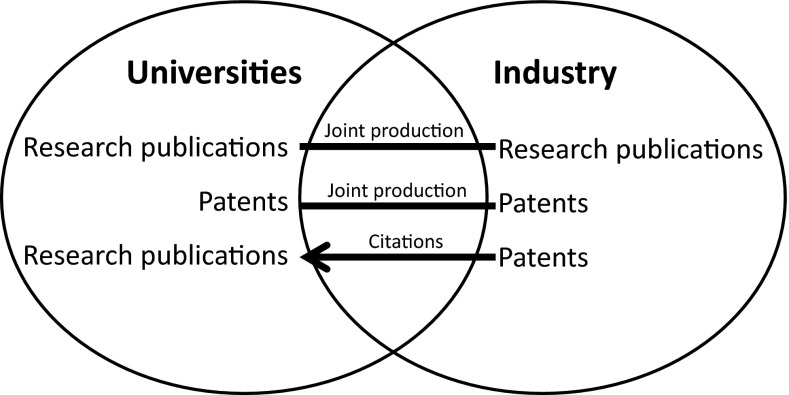
Bibliometrics-based analytical model of university-industry R&D linkages

Unlike prior validation studies by Lundberg et al. (2006), Levy et al. (2009), Wong and Singh (2013), and Yegros–Yegros et al. (2016), each dealing with UIC quantities produced by a single university, our goal is to identify general patterns across universities worldwide within the context of world university rankings. We subject UIC-based metrics to an empirical cross-validation study that incorporates several measurements associated with university–industry R&D linkages. Ideally, one would like to incorporate as much relevant information as possible, such as shared R&D facilities, joint R&D programming, research income from industry, university spin-off companies. Given the lack of such internationally comparative information, this study by necessity restricts its scope to those tangible outputs of knowledge production and absorption processes shown in Fig. 1.

Where UICs represent an output of knowledge creation processes, the knowledge absorption side is incorporated by patents and citations from patent back to research articles. These science-related *non*-*patent literature references* (NPLRs) in patents are generally seen as a proxy for science-technology linkages (Narin et al. 1997). This source provides interesting information, albeit partial conclusive results as regards to direct, causal links (Tijssen et al. 2000; Cassiman et al. 2008). Although the majority of citing patents are company-owned, some NPLRs will originate from university-owned patents. Critics correctly point out that these references may miss highly relevant knowledge flows that are more private and contract-based in nature, as well as inputs used in in-house basic research within the company (Roach and Cohen 2013). Another source of measurements are *jointly*-*applied patents with a co*-*assignee from the business sector* (e.g. Perkmann and Kathryn 2009; Briggs 2015). Hagedoorn (2003) shows that joint patenting occurs most in industries with strong intellectual property rights protection, such as the chemicals and pharmaceuticals sector. Similarly to NPLRs, these patents remain indirect indicators of linkages between scientific research and novel technologies; they and do not necessarily reflect links at the level of individuals who are often essential for effective transfer of highly-advanced technological knowledge. Of course, patent outputs may also be significantly affected by domestic framework conditions (e.g. with regards to intellectual property ownership), or other externalities beyond the control of universities.

## Data sources and methodology

Our selected sample of universities is derived from the 2014 edition of the *Leiden Ranking* (Waltman et al. 2012), which consists of the world’s 750 largest research-intensive universities according to their total research publication output in the CWTS-licensed offline version of Thomson Reuters’ *Web of Science* database. Table 1 presents the selected metrics, and their corresponding data source indicated between parentheses. Some of these metrics were extracted from existing web-based open-access sources,^6^ others were produced within the CWTS in-house information system especially for this study.

**Table 1:** Summary description of selected size-independent metrics

Metric	Description
%UIC	Share of university-industry co-publications (as a % of research publication output)
	Data source: *Leiden Ranking 2014* (publication years 2009–2012)
%MA UIC	Share of multiple-affiliation university-industry co-publications with at least one author listing a university address and a company address (as a % the total UIC output)
	Data source: *CWTS* (publication years 2009–2012; *Web of Science*)
%LOCAL UIC	Share of university-industry co-publications with at least one partner company within a 50 km range of the city in which the university is located (as a % the total UIC output)
	Data source: *U*-*Multirank 2014* (publication years 2009–2012; *Web of Science*)
%DOMESTIC UIC	Share of university-industry co-publications with at least one partner company located in the same country as the university (as a % the total UIC output); *Web of Science*
	Data source: *UIRC Scoreboard 2014* (publication years 2009–2012)
%CO-PATENT	Share of granted international patents with a co-assignee from the business sector (as % of all granted international patents)
	Data source: *U*-*Multirank 2014* (*PATSTAT* INCENTIM, Univ. Leuven; patent years 2002–2011)
%NPLR	Share of non-patent literature references, i.e. publications cited in the reference list of international patents (as a % total research publication output)
	Data source: U-Multirank 2014 (*Web of Science*;*PATSTAT* Univ. Leiden; patent and publication years 2002-2011; citing years 2002–2014)
%NPLR–HICI	Share of non-patent literature references within the world’s top 10 % most highly cited international patents across all technology areas
	Data source: *Web of Science*; *PATSTAT* Univ. Leiden (patent and publication years 2002–2011; citing years 2002–2014)

Clearly a university’s *UIC intensity* (‘ %UIC’), i.e. the share of such co-authored research publications within the organization’s total publication output, is the end result of a many inputs and processes, the determinants and contributing factors of which are based on dynamic mix of proximity-based relationships with industry and the business sector. %UIC is also one of five ‘flow’ metrics in the RIU.

The metric ‘%MA UIC’ refers to UICs where at least one of the authors has both a university affiliations and an industry affiliation, which enables us to capture parts of both the social proximity and cognitive proximity dimension of university–industry relationships. These ‘boundary spanning’ individuals are likely represent shared organizational interests or backgrounds between universities and industry of the kind that creates mutual trust and aid in effective flows of knowledge or personnel. Note than %MA UIC quantities are likely to be affected by researcher mobility patterns, institutional policies on academic appointments, as well as national laws and regulations that endorse or prohibit multiple appointments (Yegros–Yegros and Tijssen 2014).

%LOCAL UIC and %DOMESTIC UIC represent the ‘geographical proximity’ dimension, where ‘local’ is measured in terms of physical distance (research partners within a 50 km radius), while ‘domestic’ refers to partners located within the same country. For universities located at national borders, ‘local’ is not necessarily a subset of ‘domestic’. %LOCAL UIC closely relates to %MA UIC because people with simultaneous affiliations tend to have these at relatively close distance (for practical reasons of commuting). Broadening our analytical scope from ‘research’ to ‘technology’, the metric ‘%CO-PATENT’ captures ‘institutional proximity’, i.e. close relationships in terms of shared intellectual property right protection arrangements. Applying for joint patents highlights a large measure of connectedness in terms of the novel technology’s underlying R&D but also the alignment of strategic objectives to exploit the IP.

By focusing on how the scientific knowledge contributes to technological development, ‘%NPLR’ adds to ‘cognitive proximity’ dimension of our analysis. Here we assume that when the list of ‘non patent literature references’ in a patent contains a ‘citation’ to a university research publication, the technology represented by the ‘citing’ patent is now related (directly or indirectly) to the ‘cited’ research publication. Since the vast majority of patents are granted to business enterprises in manufacturing sectors, if university research publications are cited by many patents, the research is likely to have been of major relevance to technological development in the private sector. Similarly, if a university has many publications cited by patents, we may assume that it’s research portfolio was of relevance to industrial R&D and technological development. Patents are cited by subsequent patents, if the cited technology contributes to further technological development. %NPLR is the second of five ‘flow’ metrics in the TR Innovation Ranking.

Highly-cited patents are often international breakthrough technologies. %NPLR–HICI counts the number of times university research publications are cited by these ‘elite’ patents are generally seen as ‘industrially important’ technologies (Carpenter et al. 1981; OECD 2013). A high score of this metric indicates that universities are likely to have contributed knowledge of relevance to major technological developments. The NPLR data in this study were extracted from *Worldwide Patent Statistical Database* (PATSTAT) produced by the European Patent Office (EPO). PATSTAT contains patent applications that were filed at patent offices of major industrialized countries (notably USA, Japan, South Korea, Germany, China and Brazil), and major international offices such as WIPO (worldwide) and EPO itself (Europe). PATSTAT offers a broader coverage of the patent literature than the Derwent/WIPO database used by Thomson Reuters for their *Ranking of Innovative Universities*.

Patent applications that often are filed on two or three patent legislations are referred to as ‘triadic patents’ (i.e. those filed in Japan and at EPO, and also granted in the USA). Equivalent patent publications were grouped in ‘patent families’. The %CO-PATENT data were extracted by from the *PATSTAT* database at INCENTIM (Catholic University Leuven, Belgium), while %NPLR data were produced at CWTS. The NPLR-HICI relates to the 10 % most highly cited patent patents across all patent families in the PATSTAT database. Each NPLR refers either to single patents, or a single representative of patent families to remove double counting. Note that USPTO patent applications tend to contain relatively many patents from USA-based companies, each with relatively large numbers of NPLRs.

Despite the inherent limitations associated with our sample of only 750 universities, the inevitable biases of the data sources and the (still) small number of available linkage metrics, we assume that this information source provides a sufficiently robust dataset to analyze statistical relationships between UIC-based metrics and patent-based linkage metrics.

Table 2 provides summary statistics of each metric for this set of universities.^7^ Note that these comprises exclusively of size-independent metrics, thus enabling a size-corrected comparison across a diversity of universities. Some of the UIC-based metrics are close related:  %LOCAL UIC is a subset of %DOMESTIC UIC (with the exception of extremely small countries such as Singapore). %MA UIC is also closely related to %DOMESTIC UIC because people tend to have simultaneous institutional affiliations at both universities and companies if locations are within an easy travelling range—usually within the same country.

**Table 2:** Summary statistics of metrics (750 universities)

	Average	Standard deviation
%UIC	5.2	2.5
%MA UIC	.6	.6
%LOCAL UIC	18.7	13.8
%DOMESTIC UIC	60.0	21.8
%CO-PATENT	7.2	26.8
%NPLR	1.6	.8
%NPLR-HICI	4.8	2.5

By applying similar weights for each metric, RIU offers the user the benefit of transparency. The end-result, a ‘league table’, is nonetheless highly arbitrary because there is neither a theoretical justification nor a statistical rational for those weights. Data reduction techniques can help reduce or remove redundancies between metrics, where lower weights are assigned to those metrics that add little additional information. Such redundancies can be detected by applying statistical analysis to pairwise correlation coefficients between the selected metrics. Table 3 presents those coefficients, where the Pearson correlation coefficients exhibit the same pattern as the (rank-ordered) Spearman coefficients. UIC-intensity (%UIC) is correlated very significantly with  %MA_UIC and  %NPLR. In other words, a university’s UIC performance appears to be closely linked to researchers with a university affiliation and a corporate address and related to the impact of its research on technological development. Most of the other correlation coefficients among the metrics are also positive, albeit less significant. Collectively, they all reflect a broader underlying phenomenon best described as a ‘university–industry R&D linkage’.

**Table 3:** Correlations between linkage metrics (750 universities): Pearson correlation coefficients in lower-diagonal section; Spearman rank correlation coefficients in upper-diagonal section

	1	2	3	4	5	6	7
1. %UIC		**.72**	**.10**	**.42**	**.35**	**.74**	**.16**
2. %MA UIC	**.70**		**.27**	**.37**	**.25**	**.49**	.07
3. %LOCAL UIC	**.15**	**.32**		**.14**	**.08**	.04	−.06
4. %DOMESTIC UIC	**.41**	**.28**	**.21**		**.25**	**.33**	−.06
5. %CO-PATENT	**.16**	**.10**	.06	.09		**.23**	.05
6. %NPLR	**.66**	**.34**	.02	**.33**	**.19**		**.23**
7. %NPLR-HICI	**.12**	.03	**−.10**	−.07	.03	**.18**	

In bold: statistically significant at .01 (two-sided)

Principal component analysis (PCA), which draws its data from all these Pearson correlation coefficients, highlights underlying dimensions of these interrelationships.^8^ The PCA results in Table 4 shows a first component explaining 36 % of all statistical variance. The second component, accounting for an additional 17 %, mainly highlights the weak positive correlation between both NPLR-based metrics. We therefore decided to select the first component only. This component comprises university–industry R&D linkages of various kinds, reflecting in decreasing order by weight: joint knowledge creation collaboration (%UIC), social connectedness and cognitive proximity (%MA UIC), knowledge diffusion and cognitive proximity (%NPLR), and geographical proximity between partners (%DOMESTIC UIC). Of lesser relevance are ‘local’ partners, as compared to ‘domestic’, a result partly explained by the overlap with %MA UIC that may also capture geographic proximity. Similarly, %NPLR HICI is largely incorporated by %NPLR. The low weight assigned to %CO-PATENT is less easily explained and probably an outcome of its overlap with several of the other metrics.

**Table 4:** Principal component analysis and component weights (750 universities)

	Component 1 (36 % variance; Eigenvalue = 2.52)	Component 2 (17 % variance; Eigenvalue = 1.22)
%UIC	.90	.15
%MA UIC	.77	−.15
%LOCAL UIC	.35	−.65
%DOMESTIC UIC	.60	−.22
%CO-PATENT	.29	−.12
%NPLR	.74	.39
%NPLR-HICI	.13	.73

Each university’s score on this component is identical to its loading on the first component as mentioned in Table 4 (these loadings were calculated according to the regression method). In our further analysis, we will refer to this component as the *University*–*Industry R&D Linkage Index (*truncated to: U-I R&D Index). “Appendix” contains the top 100 lists according to this ranking.

## Comparing the rankings

Any index will, unavoidably, meet a challenging measurement problem: capturing the multi-dimensionality of a concept and attempting to translate it into a single metric. By its very nature, the rankings produced by a composite score rely critically on the weighting systems and can be very sensitive to variations in those weights. Table 5 presents the top 20 universities according to PCA-weighted U-I R&D Index, alongside their rankings based on an equal-weights methodology (similar to the one adopted in the *RIU ranking*). The third column contains a third variant where the weights are split equally within the two subgroups of highly related metrics: .33 each for  %MA UIC,  %LOCAL UIC and  %DOMESTIC UIC; .5 for  %NPLR and  %NPLR-HICI. Clearly, different weighting systems create different ranking positions.^9^ Removing size-dependent metrics from the ranking system, and introducing PCA-based data-driven weights, has a negative effect on MIT’s ranking, and even more so for Stanford and Harvard that have now dropped out of the top 20 (Table 8 shows their rank positions). Smaller universities move up in the ranking, especially those with relatively large numbers of UICs.

**Table 5:** Top 20 universities in the U-I R&D Index: comparing various weighting systems

	PCA-generated weights	Pre-defined equal weights	Pre-defined split weights
Eindhoven Univ. Tech.	1	29	30
Sogang Univ.	2	16	51
Delft Univ. Tech.	3	65	61
Tokyo Univ. Agr. & Tech.	4	1	1
Chalmers Univ. Tech.	5	67	64
Semmelweis Univ.	6	4	4
Technical Univ. Denmark	7	11	3
Tokyo Inst. Tech.	8	8	8
Osaka Prefecture Univ.	9	20	19
Tokyo Univ. Science	10	42	95
Massachusetts Inst. Tech.	11	44	76
Korea Adv. Inst. Sci. Tech.	12	14	11
Osaka Univ.	13	6	6
Univ Dublin Trinity Coll.	14	283	186
Keio Univ.	15	13	17
KTH Royal Inst. Tech.	16	155	193
Adv Inst. Sci. & Tech.	17	17	14
Univ. Tokyo	18	7	10
Tufts Univ.	19	15	13
Univ. Texas—Dallas	20	2	2

Such differences across rankings are inevitable, but how significant are these disparities? And how does this affect the top of the ranking distribution? Returning to our ‘target metric’, the university–industry co-publications, Table 6 summarizes the main technical differences between the various ordering metrics (either a ranking score, index score or individual scores). The overview emphasizes the disparities between the ‘broad scope’ mixed-metrics approach adopted by our U-I R&D Index, which offers the benefit of PCA-generated ‘weight optimization’ and a fully size-independent perspective.

**Table 6:** University Ranking indices and metrics: technical specifications

Ordering metric	Stock or flow metrics	Size-independent metrics	Metrics weighing	Metrics redundancy reduction
RIU	Mixed	Some	Pre-defined	No
U-I R&D Index	Flow	All	Data-driven	Yes
UIC frequency	Flow	No	–	–
%UIC	Flow	Yes	–	–

Table 7 compares the ranking positions for the 91 large research-intensive universities that feature in each of the four measure. The correlation coefficients between the rankings are all positive, and mostly very significant. Clearly all rankings capture parts of the same underlying phenomenon (loosely described here as ‘R&D linkages’, or ‘innovation’ in the case of Thomson Reuters). The RIU is more highly correlated with UIC frequency. In other words, both are oriented towards capturing volume and size. The other two metrics (U-I R&D Index and %UIC) are size-independent. Not only do these findings emphasize the major impact of including or excluding size-dependent measures in a composite index, they also question the added value of designing a composite measure in the first place. If the ‘ %UIC’ metric has such a strong relationship with *U*-*I R&D Index*, it seems more appropriate to list this metric separately rather than incorporating it in an index with a fuzzy computational origin or ambiguous meaning.

**Table 7:** Correlations between University Ranking metrics (91 universities): Pearson correlation coefficients in lower-diagonal section; Spearman rank correlation coefficients in upper-diagonal section

	1	2	3	4
1. RIU		**.43**	**.61**	**.30**
2. U-I R&D Index	**.38**		**.34**	**.84**
3. UIC frequency	**.65**	**.31**		.24
4. %UIC	.26	**.87**	.18	

In bold: statistically significant at .01 (two-sided)

Table 8 compares the RIU’s top 10 universities with their ranking position on the other three measures. The ranking similarity among the first three (size-dependent) measures is confined to the top 5 universities, mainly as a result of their large scientific publication output. MIT’s relatively low ranking on UIC output is remarkable in that respect, although this institution is among the world’s best when ranked according to the U-I R&D Index. The leading positions of Harvard, Stanford and MIT, regardless of the ranking methodology, is a testimony of their academic prowess in terms of their knowledge creation abilities and outputs relevant to research commercialization and industrial R&D.

**Table 8:** Rank positions of universities by various metrics

	RIU	U-I R&D Index	UIC frequency	%UIC
Stanford Univ.	1	25	2	34
Massachusetts Inst. Tech.	2	11	20	71
Harvard Univ.	3	39	1	203
Univ. Washington	4	121	5	129
Univ. Michigan	5	202	12	282
Northwestern Univ.	6	115	40	204
Univ. Pennsylvania	7	74	17	155
Korea Adv. Inst. Sci. Tech.	8	12	89	20
Imperial College London	9	146	9	89
Pohang Univ. Sci. Tech.	10	46	150	12

## Discussion and general conclusions

Our key question driving this validation study was: are UIC-based data suitable for assessment and comparison of universities within the context of university–industry R&D linkages? To address this question our cross-validation study examined the information value of various UIC-based measures—either as stand-alone metrics or integrated into composite indexes. We adopted Thomson Reuters’ *Ranking of Innovative Universities* (RIU) as an external frame of reference to assess their analytical relevance and statistical robustness.

When used in a stand-alone mode, UIC-based metrics should ideally comply with the newly-minted concept of ‘responsible metrics’, featuring in the recent ‘Metric Tide’ report (Wilsdon et al. 2015), and it associated list of quality criteria in which ‘robustness’, ‘transparency’ and ‘diversity’ are highlighted as key attributes. This report’s recommendations state that “indicators and metrics are based on the best possible data in terms of accuracy and scope”, “data collection and analytical processes are as open and transparent as possible, so users can test and verify results”, and “indicators and metrics to reflect and support the diversity and plurality of university performance features”.

What do UIC-based metrics represent, and how do they relate to other metrics of university ‘innovation’? Our study has demonstrated that UIC metrics are able to capture ‘diversity’ among universities: UICs represent different types of R&D-related proximity relationships, which are not easily disentangled, as well as other ‘knowledge flow’ related phenomena. UIC counts also capture size effects, and possibly even scale effects, among universities—where a few large US universities are consistently ranked among the highest worldwide.

Should one apply size-dependent or size-independent UIC-based metrics? Including size-dependent measures has major implications for positions of universities in the Thomson Reuters’ ranking. Many less prolific universities, i.e. with smaller quantities of research publications and/or patents, are significantly disadvantaged. Our findings show that this is particularly detrimental for the high-performing Japanese universities as well as some Western European universities, which are all ranked in the top 25 according to the *U*-*I R&D Linkage Index* (see Table 9). Clearly, whether or not to incorporate a university’s size is one of the key determinants of its ranking position. The same applies to single UIC metrics: applying the absolute number of UICs produces a completely different picture compared to the share of UICs in university’s publication output. While offering the advantage of computational transparency, their reliance on a single source of information reduces their analytical scope. UICs alone are clearly an insufficient measure of university–industry R&D linkages. These co-authored publications are just one of many measurable linkages between universities and the business sector.

Does that make data-heavy, multiple-metrics indexes superior analytical tools? What are the (dis)advantages when several metrics are combined or integrated into a composite measure? Composites clearly offer ways of incorporating a wide range of sources and metrics, thereby reducing negative effects of source-specific and country-specific differences between universities, and thus providing a more balanced and robust measure suited for comparative purposes. Composite measures, partially based on UIC-metrics, are therefore a superior proxy, provided both absolute and relative size needs are factored in. Although this might enable us to identify top ranked ‘powerhouse’ universities, no index alone can provide a fully adequate assessment of an institution’s performance. Any index-based university ranking will still depend significantly on the selected metrics, the computational method that was applied to assembled in a composite measure.

This tradeoff between single-metrics and multiple-metrics needs careful navigation in search of an optimized approach. Inevitably, differences are to be expected and significant discrepancies between ranking positions may easily arise. Usually as a result of an interplay between the choice of metrics, the computational methods applied, and because of differences between information sources.

Our sensitivity tests indicate that the various UIC-based metrics and indexes are not consistent proxy measures: in most cases a university’s spread of ranking positions across the between the various rankings is simply too large. Although both index-based rankings in this study are, in a sense, useful representatives of U-I R&D linkages, there is no formalized rationale or intuitive heuristic to opt for either as ‘the best’ representation. Nor is there a ‘one-size-fits-all’ measurement model. Our findings also highlight both conceptual and computational weaknesses in the RIU. Such top-down producer-driven rankings, with a single composite score based on questionable weighting systems, are suboptimal data reduction tools. Clearly, the trade-off between data reduction and generating meaningful outcomes is problematic and in effect disqualifies these league table rankings as representing ‘best practice’.

Can one develop a ‘good practice’ to develop more robust and meaningful university ranking systems? Based on this study’s outcomes, we recommend a bottom-up ‘scoreboard’ approach without those problematic data reduction constraints. This user-driven approach has already been adopted in the *Leiden Ranking* and *U*-*Multirank*, both ‘open access’ information tool where: (1) no composite measures and weighting systems of metrics are implemented, (2) computational procedures and information sources are more transparent, (3) size-dependent metrics are kept separate from size-independent metrics, (4) performance metrics are selected according to proximity relationships between universities and industry. Users should be able to (de)select metrics of their choice according to their own selection criteria and analyze the scoreboard data anyway they like to satisfy their information requirements.

Irrespective of the measurement model, choice of metrics, and the type of ranking system, the availability of such information has opened up a new comparative framework with implications for various major stakeholders.^10^ First, the ranking producers themselves: here we expect to see follow-up publications of ranking data. On June 14th, 2016 Thomson Reuters has published the list of ‘Europe’s top 100 innovative universities’.^11^ More university rankings dealing with aspects related to ‘innovation’ (or related topics such as ‘entrepreneurship’ or ‘industry orientation’) will surely appear in the years to come. Secondly, the universities where one may expect to find the same institutional response as in the case of other rankings: high scores are likely to be publicly acknowledged on university websites or in press releases; low scores will be either ignored or perhaps used in management decision-making on the strategic development of a university. The popular press is a third stakeholder: any newsworthy information introduced by university rankings is of interest. More rankings creates more opportunities for news articles and blog posts.

These new ranking systems on ‘innovation’ may also create applications in government policy making domains. Although we are unaware of such policy impacts to this date, we may speculatively assume that such rankings will indirectly affect policy making at the national or regional level. Those impacts could emerge in terms of offering a new source of evidence for policy debate or raising the awareness of such performance measures for policy recommendations. It seems much less likely that rankings, given their still questionable robustness, will be used to support resource-allocation decisions—either to address perceived shortcomings in university–industry R&D linkage relationships or to boost the performance of individual ‘innovation-oriented’ universities.

## Appendix

See Table 9.

**Table 9:** Top 100 universities according to University-Industry R&D Linkage Index (*with ranking positions in Thomson Reuters RIU added between parentheses*)^*b*^

1		Eindhoven University of Technology	Netherlands
2		Sogang University	South Korea
3	(73)	Delft University of Technology	Netherlands
4		Tokyo University of Agriculture and Technology	Japan
5		Chalmers University of Technology	Sweden
6		Semmelweis University of Budapest	Hungary
7	(43)	Technical University of Denmark	Denmark
8	(51)	Tokyo Institute of Technology	Japan
9		Osaka Prefecture University	Japan
10		Tokyo University of Science	Japan
11	(2)	Massachusetts Institute of Technology	United States
12		Korea Advanced Institute of Science and Technology	South Korea
13	(18)	Osaka University	Japan
14		Trinity College, Dublin	Ireland
15	(58)	Keio University	Japan
16		KTH Royal Institute of Technology	Sweden
17		Advanced Institute of Science and Technology	Japan
18	(24)	University of Tokyo	Japan
19	(34)	Tufts University	United States
20		University of Texas, Dallas	United States
21	(39)	Tohoku University	Japan
22		Grenoble INP	France
23		Tokyo Medical and Dental University	Japan
24		Osaka City University	Japan
25	(1)	Stanford University	United States
26		University of Medicine and Dentistry of New Jersey	United States
27		University of Copenhagen	Denmark
28	(23)	Georgia Institute of Technology	United States
29		Rensselaer Polytechnic Institute	United States
30		Chiba University	Japan
31		Drexel University	United States
32		Juntendo University	Japan
33		Aalto University	Finland
34		Aalborg University	Denmark
35		Rockefeller University	United States
36		Kanazawa University	Japan
37		Shinshu University	Japan
38		University of Tokushima	Japan
39	(3)	Harvard University	United States
40	(62)	Hanyang University	South Korea
41		Thomas Jefferson University	United States
42		University of Twente	Netherlands
43		Technical University of Graz	Austria
44		Budapest University of Technology and Economics	Hungary
45		Wageningen University and Research Centre	Netherlands
46	(12)	Pohang University of Science and Technology	South Korea
47	(31)	Seoul National University	South Korea
48		University of Gothenburg	Sweden
49	(13^a^)	University of California, San Diego	United States
50	(13^a^)	University of California, San Francisco	United States
51		Kobe University	Japan
52		Karolinska Institute	Sweden
53		University of Maryland, Baltimore	United States
54		Uppsala University	Sweden
55	(81)	Kyushu University	Japan
56	(22)	Kyoto University	Japan
57		Yamaguchi University	Japan
58		University of Texas Health Science Center, Houston	United States
59		Kitasato University	Japan
60		Yokohama City University	Japan
61		Waseda University	Japan
62	(84)	Korea University	South Korea
63		University of Massachusetts Medical School	United States
64	(36)	Yonsei University	South Korea
65	(98)	Hokkaido University	Japan
66		Linköping University	Sweden
67	(52)	Friedrich-Alexander-Universität Erlangen-Nürnberg	Germany
68		St Louis University	United States
69		Lund University	Sweden
70		Georgetown University	United States
71		Saarland University	Germany
72		Ajou University	South Korea
73		Gunma University	Japan
74	(19)	Johns Hopkins University	United States
75	(9)	University of Pennsylvania	United States
76	(66)	Sungkyunkwan University	South Korea
77		University of Eastern Finland	Finland
78		Hiroshima University	Japan
79		Kinki University	Japan
80	(41)	University of Utah	United States
81		Yeshiva University	United States
82	(17)	Duke University	United States
83		University of Southern Denmark	Denmark
84	(82)	Case Western Reserve University	United States
85		University of Basel	Switzerland
86		National Chiao Tung University	Taiwan
87		Humboldt University of Berlin	Germany
88	(59)	Boston University	United States
89		Gifu University	Japan
90	(47)	Baylor College of Medicine	United States
91		Heinrich Heine Univ Düsseldorf	Germany
92	(89)	Nagoya University	Japan
93		University of Colorado, Denver	United States
94	(37)	Swiss Federal Institute of Technology Zurich	Switzerland
95	(96)	Freie Universität Berlin	Germany
96		Cranfield University	United Kingdom
97	(14)	University of Southern California	United States
98		Kumamoto University	Japan
99	(13^a^)	University of California, Los Angeles	United States
100	(56)	Carnegie Mellon University	United States

^a^Ranking position refers to the University of California system

^b^ *Source*: https://www.timeshighereducation.com/worlds-most-innovative-universities-2015-results (accessed on June 24, 2016)

## Footnotes

1 The eight selected metrics are: total number of research articles in Thomson Reuters *Web of Science* database (‘Publication Volume’); total number of patents filed at the *World International Patent Organisation*—WIPO (‘Patent Volume’); the ratio of those patent applications to WIPO granted patents (‘Patent Success’); patent to patent citation impact (‘Patents Citations’); Relative citation impact (‘Patent Citation Impact’); citation from patents to research publications (‘Patent to Article Citation Impact’); citation impact from industry-produced publications (‘Industry Article Citation Impact’); percentage of university-industry co-authored publications (‘Percent of Industry Collaborative Articles’).

2 With the exception of ‘Patent Citation Impact’ and ‘Patents Citations’, where both metrics account for a 50 % fraction of their total weight.

3 The top 100 list of RIU universities is available at the following website: http://www.reuters.com/article/idUSL1N11K16Q20150915.

4 The UIC indicator was removed from the 2016 edition of the *Leiden Ranking* but will reappear in an associated information tool that will be launched by CWTS in 2017.

5 For more background information about these sources and their range of metrics, we refer to the following webpages with relevant technical details: http://www.leidenranking.com/information/; http://www.umultirank.org/#!/about/methodology/approach-to-ranking.

6
*U-Multirank*, *Leiden Ranking* and *UIRC Scoreboard 2014* (www.cwts.nl/UIRC2014).

7 With the exception of %MA UIC, all metrics show the normal distribution of score across the selected set of universities. The values for asymmetry and kurtosis between −2 and +2 are considered acceptable in order to prove normal univariate distribution (George and Mallery 2010). The corresponding values for %MA-UICs are 3.6 and 23.

8 PCA transforms a number of correlated variables into a smaller number of uncorrelated variables called principal components. These components are linear combinations of the original variables weighted by their contribution to explaining the variance in a particular orthogonal dimension. The first component accounts for as much of the variance in the data as possible, where each succeeding component accounts for as much of the remaining variance as possible.

9 This study’s aim was to illustrate the range of various weighting options, and their consequences for ranking positions. Hence, we refrained from conducting a sophisticated sensitivity analysis, with repeated random sampling of weights, to assess the statistical robustness of these three rankings.

10 This type of comparative information on universities might also be of some relevance for small, specific groups of prospective (industrial) PhD students or R&D companies cooperating with universities, but we consider these to be ‘minor stakeholders’ compared to the other three stakeholder groups.

11
http://www.reuters.com/article/us-innovative-stories-europe-idUSKCN0Z00CT.
